# Acute sonographic changes in common carotid artery after NESA neuromodulation intervention in healthy adults: a randomized controlled clinical trial

**DOI:** 10.3389/fnins.2025.1526236

**Published:** 2025-04-28

**Authors:** Isabel Mínguez-Esteban, Mónica De la Cueva-Reguera, Vanesa Abuín-Porras, Carlos Romero-Morales, Jaime Almazán-Polo, María Bravo-Aguilar

**Affiliations:** Faculty of Sport Sciences, Universidad Europea de Madrid, Villaviciosa de Odón, Madrid, Spain

**Keywords:** NESA neuromodulation, autonomic nervous system, common carotid artery (CCA), vascular ultrasound Doppler, vascular ultrasonography, lumen diameter (LD), peak systolic flow velocity, electrical nerve stimulation

## Abstract

**Introduction:**

The endothelium plays a key role in vascular health, and its dysfunction is a major risk factor for cardiovascular diseases (CVD). Non-invasive neuromodulation techniques, such as NESA, aim to improve vascular tone and function by targeting the autonomic nervous system. However, evidence regarding their acute vascular effects is still limited.

**Methods:**

A randomized controlled trial was conducted with 40 participants divided into NESA (*n* = 20) and placebo (*n* = 20) groups. Both groups underwent 20-min interventions. Sonographic assessments of the left CCA, including lumen diameter (LD), intima-media thickness (IMT), and peak systolic velocity (PSV), along with blood pressure (BP) and heart rate (HR), were performed before and immediately after the intervention.

**Results:**

Significant increases in LD and cross-sectional area (CSA) were observed in the NESA group compared to placebo (*p* < 0.001), alongside a decrease in IMT (*p* < 0.05). HR showed a significant reduction post-intervention in both groups, with a more pronounced effect in the NESA group (*p* = 0.001). No significant changes were found in BP or PSV.

**Discussion:**

The findings demonstrate that NESA neuromodulation induces immediate changes in vascular parameters, including increased LD and CSA and decreased IMT. These results highlight measurable acute vascular effects in healthy individuals following NESA intervention.

## 1 Introduction

The endothelium plays a vital role in regulating vascular response by releasing vasoactive substances that act on vascular smooth muscle, promoting vasodilation—a critical mechanism for maintaining proper blood flow and pressure. The disruption of the normal vascular response is strongly associated with the development of cardiovascular disease (CVD) risk factors, such as hypertension and hypercholesterolemia, and has also been linked to conditions like diabetes, obesity, and heart failure, highlighting its pivotal role in the onset and progression of CVD (ter Maaten et al., 2016; Shi and Vanhoutte, 2017; Limberg et al., 2022; Sabe et al., 2022).

The autonomic nervous system (ANS) is considered a key factor that affects the behavior of the endothelial function by controlling lumen diameter (LD), blood flow, and the release of key signaling molecules, such as nitric oxide (NO). Vasodilation and vasoconstriction through the sympathetic and parasympathetic innervations keep the homeostasis related to the blood pressure and vascular resistance (Schultz, 2009; Gaertner et al., 2023). Thus, evidence suggests that an imbalance of the ANS could contribute to the development of vascular diseases, as vascular integrity is essential for maintaining efficient blood circulation and preventing endothelial-related pathologies (Bruno et al., 2012; Waclawovsky et al., 2021).

Recent advancements in approaches which target the ANS have been shown in the last decade, particularly those related to cardiovascular and psychiatric conditions (Wang et al., 2021). For example, electrical vagus nerve stimulation has reported benefits in the reduction of the inflammation and improving the heart rate variability (Salavatian et al., 2024). Regarding the influence of direct nervous system stimulation through electrical currents, novel devices and techniques have emerged. NESA is a non-invasive, affordable, and portable nerve stimulation technique that uses sub-sensory microcurrents below 1 mA for superficial neuromodulation (Medina-Ramírez et al., 2021; García et al., 2022; Teruel-Hernández et al., 2023). By stimulating peripheral nerve terminals, it targets the ANS to regulate the structures it governs, such as the vascular system, particularly by modulating sympathetic nervous activity (Medina-Ramírez et al., 2021). While the exact mechanisms remain under investigation, emerging evidence suggests that microcurrent stimulation may increase catecholamine secretion, such as noradrenaline, from postganglionic sympathetic neurons, potentially affecting vascular responses like vasomotor tone and arterial stiffness through the activation of autonomic pathways (Thijssen et al., 2011; Kolimechkov et al., 2022). Unlike transcutaneous vagus nerve stimulation, which typically involves higher-intensity currents and direct stimulation of the vagus nerve at its auricular territory, NESA applies diffuse stimulation across multiple points, emphasizing its unique approach to neuromodulation.

Ultrasound imaging of the common carotid artery (CCA) offers a safe and accessible method for assessing vascular responses, including lumen diameter (LD), intima-media thickness (IMT), and pulse wave velocities such as peak systolic velocities (PSV). CCA diameter often increases to compensate for arterial wall thickening, helping maintain normal blood flow despite higher IMT. This adaptation may be influenced by the parasympathetic system, suggesting an interaction between autonomic function and vascular dynamics (Chironi et al., 2009).

Therefore, the aim of the present study is to evaluate the acute effect immediately after the application of the NESA treatment in healthy participants in vascular sonographic variables measured in the left CCA such as the LD, IMT, PSV, as well as hemodynamic variables, including systolic (sBP) and diastolic (dBP) blood pressure and heart rate (HR). We hypothesize that NESA application will directly influence changes in the hemodynamic variables and left CCA sonographic variables compared to the Placebo group. This premise is based on the hypothesis that non-invasive NESA neuromodulation will influence the autonomic regulation of vascular responses through the stimulation of the ANS.

## 2 Materials and methods

### 2.1 Design

A randomized controlled trial followed the Consolidated Standards of Reporting Trials (CONSORT) checklist and was registered on ClinicalTrials.gov under the identifier NCT06320171. The research complied with the ethical standards outlined in the Declaration of Helsinki and relevant regulations governing human experimentation. Ethical approval was granted by the institutional research committee of Universidad Europea de Madrid under the reference code CIPI/2024.481.

### 2.2 Sample size calculation

The sample size was determined through an a priori power analysis for an ANOVA with repeated measures, between factors, using G^*^Power 3.1.9. (GPower ©, University of Düsseldorf, Düsseldorf, Germany), based on a directly specified moderate effect size (*f* = 0.4), given the absence of prior specific data for the intervention. The analysis assumed a moderate effect size (*f* = 0.4), a significance level of α = 0.05, and a statistical power (1 – β) of 0.8 (Cohen, 2013; Lakens, 2013). A correlation among repeated measures of 0.5 was also assumed, with an equal number of measurements per group (two groups with two measurements each). Based on these parameters, the required total sample size was calculated to be 40 participants, equally divided between the intervention group (NESA, *n* = 20) and the Placebo group (*n* = 20). This sample size ensures adequate statistical power to detect significant differences between groups, with an actual power of 0.8127.

### 2.3 Participants

A total sample of forty participants (*N* = 40; mean age 27 ± 7 years) consisting of university students, faculty, and staff, were recruited for the study (Stefánsson et al., 2006). To ensure sample homogeneity and reduce age-related variability, participants were selected within a similar age range. Prior to entering the study, all participants received information about the study and signed a consent form to participate. The study subjects were divided into two equally distributed groups (A: NESA = 20; B: Placebo = 20) and were blinded to the study group allocation. Participants in the Placebo group underwent the same procedural phases as those in the intervention group. However, for the Placebo group, the device was configured in “Placebo” mode where the device remains on but is not emitting current during the intervention time. All participants received identical information about the procedure, regardless of group assignment. Participants were randomly assigned to the Placebo or Intervention group using the opaque envelope system, where the intervention group was assigned as “Group A” and the Placebo as “Group B”.

The inclusion criteria for participants were: (a) healthy adult males and females, (b) between 18 and 45 years of age, (c) no previous experience receiving NESA therapy. Subjects were excluded if they had a history of cardiovascular disease, carotid echography not feasible, metabolic disease, autoimmune disease, neurological disease, hypertension, congenital abnormalities or inability to understand the intervention protocol, as well as having taken analgesics or anti-inflammatory drugs 1 week prior to participation. Participants were also informed that they had to attend the evaluation in fast conditions (08:30–10:30), without taking caffeine, theine or exercising before the intervention.

### 2.4 Automatic BP measurement

BP was assessed using an automated blood pressure monitor (OMRON M2 Basic HEM-7121J-E, OMRON Healthcare Europe B.V., Netherland) by the same evaluator before and at the end of the intervention. With the patient lying supine in a 30° recumbent position, left arm blood pressure was assessed in all participants after 15 min resting. Three measurements were taken to determine the average of sBP and dBP assessments (James and Gerber, 2018).

### 2.5 NESA and placebo group intervention

During the intervention, patients were seated comfortably on a sofa with their backs supported. NESA, a portable, noninvasive neuromodulation device, delivered low-frequency (1.3–14.28 Hz), low-intensity (0.1–0.9 mA), low-voltage (±3 V) microcurrents through 24 electrodes placed on the distal nerve endings of the wrists and ankles (six electrodes per limb) (Teruel-Hernández et al., 2023). Through the simultaneous coordination of 24 electrodes by means of multipolar current (six per limb placed through anklets and gloves) and an additional directional electrode at the C7 spinous process (NESA device Program 7), the distributed placement and the ultra- low electrical parameters of the microcurrents produce a systemic, sub-sensory effect, rather than a localized activation of specific muscles or nerve areas, potentially engaging the autonomic nervous system through the hypothesized cutaneous distribution (Glatte et al., 2019).

Direct stimulation of the autonomic nervous system was achieved using Program 7 (P7) of the device, with a directional electrode positioned at the level of the C7 spinous process. P7 delivered a biphasic polarity current with oscillatory frequencies ranging from 1.92 to 14.29 Hz and variable intensities between 0.1 and 0.9 mA. Due to the specific parameter configuration, the electrical stimulus was imperceptible to the patient (García et al., 2022; Teruel-Hernández et al., 2023). The NESA group (*n* = 20) underwent a 20-minute intervention protocol while sitting reclined on a couch that facilitated the placement of the NESA electrodes. After this application, after 10 min of removing the material and resting, the patients returned to their initial position on the stretcher, where BP and ultrasound variables were immediately re-evaluated in the same position previously described. The Placebo group (*n* = 20) followed the same procedure, except that no current was applied during their 20-min session.

### 2.6 Sonographic assessment of left CCA

Ultrasound evaluation of the common carotid artery was performed using established guidelines for reliable evaluation (Thomas et al., 2015; Murray et al., 2018). All ultrasound evaluations were carried out by the same sonographer with more than 5 years of experience in musculoskeletal and vascular ultrasound before and after the intervention in both study groups. For the evaluation of the CCA, the evaluator positioned himself at the head of the subject, slightly lateral to them, in a wheeled chair to facilitate movement and comfort. The ultrasound used was a SAKURA P10 SonoScape with a 12L-B Linear Array transducer and 17-3 MHz frequency, set to a standard depth of 4 cm, with a pixel size of 0.11 mm. The zoom function was not used to avoid loss of external reference points and resolution, and subsequent video sequences and images were evaluated (Chironi et al., 2009).

**Figure 1:**
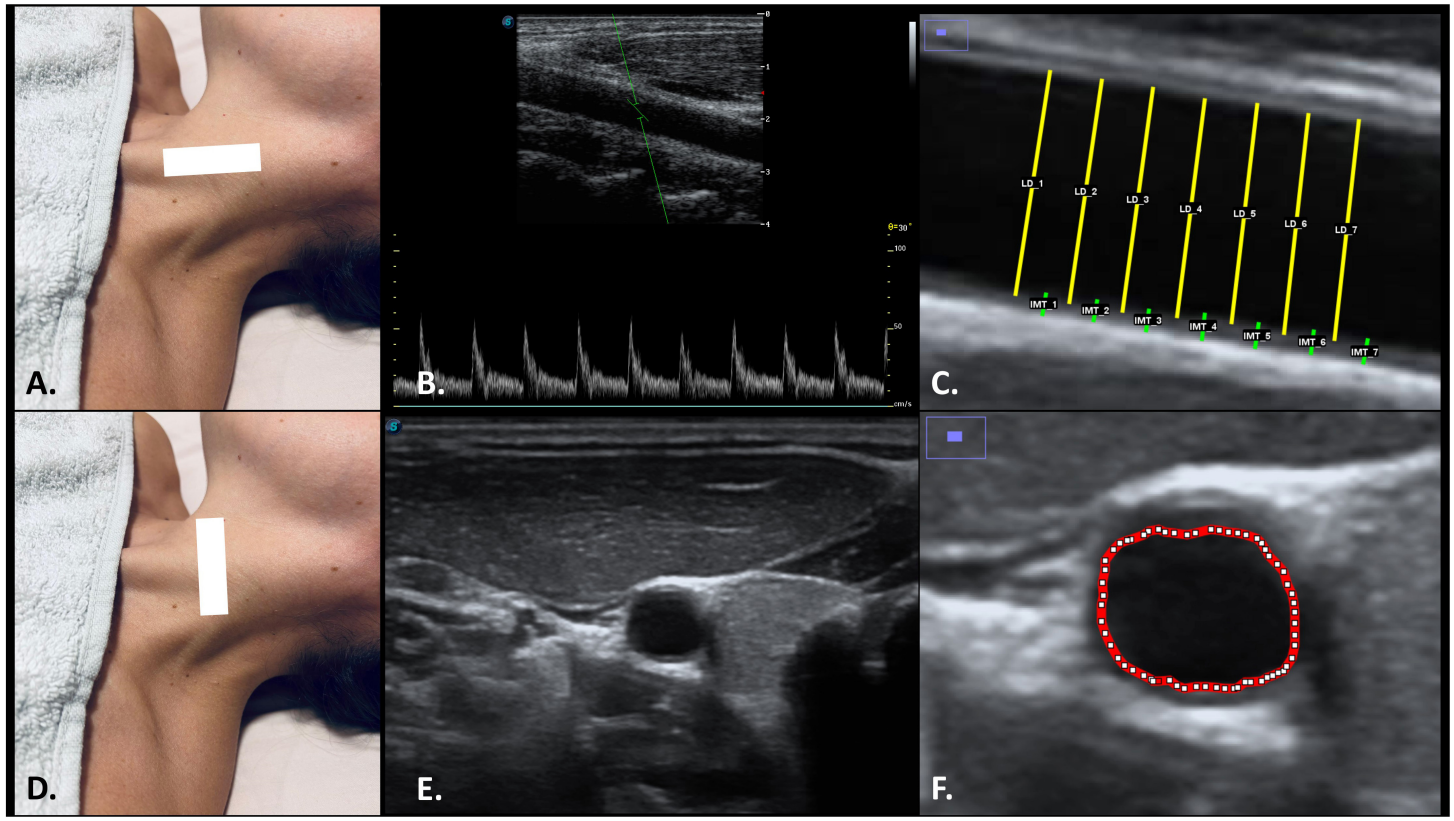
Ultrasonographic and ImageJ assessment of left CCA in long axis view **(A)** for PSV **(B)**, LD and IMT **(C)**; as well as in short axis **(D)** view for CSA **(E, F)**. The probe was placed at the level of the left CCA using the SCM muscle as an acoustic window **(A, B)**. Longitudinal sections on the left CCA **(B, C)** allowed to determine the peak systolic velocities (PSV) and the changes in the LD and IMT, while the transverse sections **(E, F)** allowed to determine the CSA. Abbreviations: CCA, common carotid artery; CSA, cross-sectional área; IMT, intima–media thickness; LD, lumen diameter; PSV, peak systolic velocities; SCM, sternocleidomastoid muscle.

Subjects were positioned as previously described for BP assessment, lying supine with the table backrest reclined at a 30° angle. The head was rotated 45° to the right to facilitate probe placement for the evaluation of the left CCA ultrasound, and a pillow was placed under the neck to control cervical extension. Ultrasound assessment of the left CCA was performed after BP measurement, both before the intervention and 10 minutes after its completion, during the process of removing the NESA materials. CCA was analyzed 1 centimeter before entering the bulb and divided in the internal and external carotid segments. The ultrasound probe was placed transversely to the area to be explored to identify the division of the carotid artery. Posteriorly, the probe was modified to obtain long-axis images in the position of the artery. Once the corresponding image of the CCA was identified, three images were taken in short transverse axis for subsequent area section analysis (Tahmasebpour et al., 2005). Subsequently, longitudinal measurements of the carotid artery were taken using spectral Doppler mode to obtain a graphical representation of the pulse wave velocity, recording the peak systolic velocities (PSV). The sample volume was set to a size of 2 mm and positioned at the center of the vascular lumen. The adjustments made for the calculation of the PSV were based on the technical recommendations of Thomas et al. (2015) for carotid duplex ultrasound evaluation. Ultrasound probe was tilted distally 30° by resting on the left lateral side of the neck, at the height of the thyroid gland position. The angle corrections were then made with the steering function of the ultrasound incorporating an angle of 20°, so that all evaluations were made between 30°-60° with respect to the direction of the distal—proximal carotid flow. Ten peak systolic measurements were recorded to calculate the average PSV. Lastly, three longitudinal section images and videos were stored, recording the images for the external measurement of the arterial lumen diameter between the intima to intima near and far walls of the CCA, respectively (double-line sign). For proper ultrasound evaluation, the probe was held with both hands to apply the least possible pressure to visualize the CCA, as well as applying a high amount of ultrasound gel to reduce bias due to excessive compression. Images and videos of the left CCA were taken and analyzed at the peak of greatest dilation with respect to the arterial pulse. All data obtained from the left common carotid artery were stored in DICOM format and analyzed using the external free open-source ImageJ—Fiji (U.S. National Institutes of Health; Bethesda, Maryland, USA). The average of 7 tracings was calculated for the intima-to-intima distance for the carotid artery diameter (LD), as well as the intima-media distance for the thickness (IMT) (Figure 1) (Wikstrand, 2007; Murray et al., 2018). All measurements were performed on a 1 cm wide section, 2 cm prior to the proximal division of the artery. Finally, the absolute difference [Abs. Dif._LD (mm) = LD Post session – LD Baseline] and relative difference [Rel. Dif._LD (%) = LD Post session – LD Baseline/LD Baseline] were calculated to determine the proportion of change in the left CCA between interventions.

### 2.7 Statistics

The statistical analysis was performed by Jamovi software (v.2.3, Jamovi project). Firstly, Shapiro-Wilk test was employed in order to check the normality assumption of the variable's distribution. Secondly, an independent *T*-test was used to compare the Abs. Dif._LD and Abs. Dif._LD variables between NESA and Placebo group. The effect size for these comparisons was calculated by Cohen's d, which are interpreted as follows: small effect (*d* = 0.2), medium effect (*d* = 0.5), and large effect (*d* = 0.8). A repeated measures ANOVA test was employed to examine the effects of treatment (NESA vs. Placebo) and time (pre-test and post-test) on the dependent variables. The partial eta-squared (η^2^*p*) was used as a measure of effect size in the ANOVA, with values interpreted based on conventional thresholds: small effects (η^2^*p* = 0.01), medium effects (η^2^*p* = 0.06), and large effects (η^2^*p* = 0.14).

The intraclass correlation coefficient (ICC) was calculated using a two-way mixed-effects model with absolute agreement, based on two intra-rater and two inter-rater measurements, along with the associated 95% confidence interval, to assess parameter reliability. ICC values were categorized as poor (< 0.40), fair (0.40–0.59), good (0.60–0.74), and excellent (0.75–1.00) (Hallgren, 2012). ICC values exceeding 0.90 were considered indicative of highly reliable clinical measurements (Portney and Watkins, 2009). Additionally, the standard error of measurement [SEM = SDpooled × √(1 – ICC)] and minimum detectable change (MDC = √2 × 1.96 × SEM) were calculated (Furlan and Sterr, 2018). SEM reflects random score variation in the absence of actual change, while MDC represents the smallest detectable change considered significant (Furlan and Sterr, 2018).

## 3 Results

The sociodemographic data (Table 1) revealed no significant differences between NESA and Placebo group in terms of age, height, sex distribution or BMI. However, a significant difference was found in weight, with the NESA group reporting a higher mean weight compared to Placebo (*p* = 0.024).

**Table 1:** Sociodemographic data of the sample.

**Data**		**NESA (*n =* 20)**	**Placebo (*n =* 20)**	***P*-value**
**Age**, ***y***		25 ± 10	24 ± 4	0.149^*^
**Weight, kg**		73.8 ± 11.0	66.2 ± 9.70	**0.024** ^†^
**Height, m**		1.72 ± 0.07	1.69 ± 0.08	0.197^*^
**BMI, kg/m** ^**2**^		24.9 ± 3.54	23.1 ± 2.17	0.059^*^
**Sex**	**Women**	12 (57.1%)	9 (42.9%)	0.342^‡^
		**Men**	8 (42.1%)		11 (57.9%)	

BMI, *body mass index*.

^*^ Median ± intercuartilic range and range (min – max) were used according to Shapiro Wilk test for non-parametric data distribution.

^†^U-Mann Whitney for paired independent samples was used according to the non-parametric distributions.

^‡^Frequency and relative percentage (%) as well as well as Chi-squared test (χ 2) were used.

For all analyses, p < 0.05 (for a confidence interval of 95%) was considered statistically significant (**bold**).

**Figure 2:**
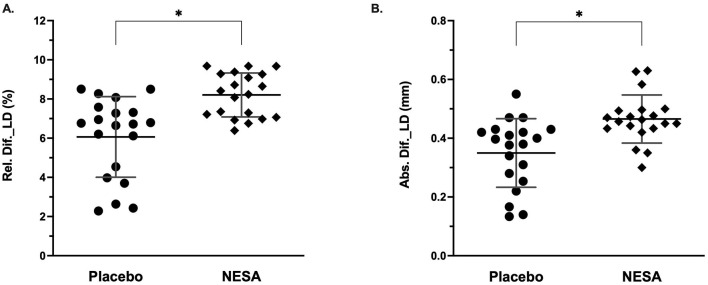
Lumen diameter [**(A)** Relative, %; **(B)** Absolute, mm] differences between NESA and Placebo. ^*^Significant differences were observed between groups.

Considering the intrasubject effects (Table 2), our results reported significant differences in several outcomes between the NESA and Placebo. For the CCA_CSA measure, both groups exhibited improvements, with a significant time effect and interaction between treatment and time favoring the NESA group (*p* = 0.002, *p* = 0.001, respectively). Similarly, for LD, a strong time effect and a treatment-time interaction were observed, both in favor of NESA (*p* = 0.001), as well as the IMT variable showed a moderate interaction effect in time and in treatment-time in the decrease of NESA vs. Placebo (*p* = 0.024 and p = 0 0.025, respectively). No significant differences were noted for PSV or dBP, but a trend toward significance was seen for sBP (*p* = 0.066). Finally, HR showed a significant reduction in the NESA group, with a notable time effect (*p* = 0.001), though the treatment-by-time interaction did not reach significance. Abs. Dif._LD (*p* = 0.001, Cohen's *d* = 1.15) and Rel. Dif._LD (*p* = 0.001, Cohen's *d* = 1.33) variables showed significant differences between groups in favor of the NESA group with respect to the Placebo group (Figure 2).

**Table 2:** Sonographic vascular variables of left CCA, blood pressure and heart rate differences between groups.

**Variable**		**NESA (*n =* 20)**	**Placebo (*n =* 20)**	**Intrasubject effects**	
				**Time value [*F* (Df); *P* (η^2^*p*)]**	**Treatment × time [*F* (Df); *P* (η^2^*p*)]**
**CSA, mm** ^**2**^	Baseline	19.8 ± 4.0	19.5 ± 2.4	F_(1, 38)_ = 11.4; *P =* **0.002** (0.230)	F_(1, 38)_ = 12.2; P =**0.001** (0.243)
	Post-test	22.9 ± 4.2	19.4 ± 1.6		
**LD, mm**	Baseline	5.7 ± 0.9	5.8 ± 0.7	F_(1, 38)_ = 655.1; *P =* **0.001** (0.945)	F_(1, 38)_ = 13.1; P =**0.001** (0.257)
	Post-test	6.17 ± 0.9	6.19 ± 0.9		
**IMT, mm**	Baseline	0.45 ± 0.25	0.44 ± 0.24	F_(1, 38)_ = 5.55; *P =* **0.024** (0.127)	F_(1, 38)_ = 5.48; P =**0.025** (0.126)
	Post-test	0.42 ± 0.22	0.44 ± 0.24		
**PSV, cm/s**	Baseline	30.5 ± 9.6	28.0 ± 7.3	F_(1, 38)_ = 0.091; *P =* 0.763 (0.002)	F_(1, 38)_ = 0.001; *P =* 0.974 (0.001)
	Post-test	31.0 ± 9.5	28.5 ± 8.4		
**sBP**	Baseline	117 ± 10.9	113 ± 12.9	F_(1, 38)_ = 3.571; *P =* 0.066 (0.086)	F_(1, 38)_ = 0.099; *P =* 0.754 (0.003)
	Post-test	113 ± 10.3	110 ± 14.1		
**dBP**	Baseline	67.5 ± 7.8	66.5 ± 7.4	F_(1, 38)_ = 0.232; *P =* 0.632 (0.001)	F_(1, 38)_ = 0.025; *P =* 0.873 (0.001)
	Post-test	67.0 ± 6.5	65.5 ± 11.9		
**HR, bpm**	Baseline	65.0 ± 13.6	64.8 ± 8.4	F_(1, 38)_ = 43.28; *P =* **0.001** (0.533)	F_(1, 38)_ = 2.09; *P =* 0.157 (0.052)
	Post-test	57.5 ± 11.1	60.0 ± 6.5		

CSA, cross – sectional area; bmp, beats per minute; dBP, diastolic blood pressure; IMT, intima-media thickness; LD, lumen diameter; PSV, blood velocities at systolic peaks; sBP, systolic blood pressure.

For all analyses, p < 0.05 (for a confidence interval of 95%) was considered statistically significant (**bold**).

The reliability analysis of vascular ultrasound variables was conducted on 10 participants (5 from the NESA and 5 from the Placebo, including the first 3 men and 2 women from each group), demonstrating excellent reliability across all variables (Table 3). The analysis demonstrated consistently high intraclass correlation coefficients (ICC ≥ 0.996), accompanied by low SEM and MDC, supporting the reliability and methodological precision of the ultrasound assessments.

**Table 3:** Means, standard deviation, intraclass correlation coefficient, standard error measurement and minimum detectable change of left CCA ultrasonographic variables.

**Variable**	**Test 1 (Mean ±SD)**	**Test 2 (Mean ±SD)**	**Test 1–2 SE**	**ICC (95% CI)**	**SEM**	**MDC 95%**
**Baseline CSA, mm** ^**2**^	22.24 ± 3.49	22.12 ± 3.51	1.107	0.999 (0.999 – 0.999)	0.111	0.466
**Post-test CSA, mm** ^**2**^	22.81 ± 3.80	22.78 ± 3.78	1.199	0.999 (0.999 – 0.999)	0.111	0.466
**Baseline LD, mm**	6.12 ± 0.54	6.09 ± 0.54	0.17	0.997 (0.988 – 0.999)	0.030	0.241
**Post-test LD, mm**	6.32 ± 0.65	6.32 ± 0.67	0.21	0.996 (0.985 – 0.999)	0.042	0.286
**Baseline IMT, mm**	0.37 ± 0.14	0.37 ± 0.12	0.04	0.947 (0.785 – 0.987)	0.030	0.243
**Post-test IMT, mm**	0.36 ± 0.14	0.36 ± 0.12	0.04	0.935 (0.732 – 0.984)	0.033	0.255
**Rel. Dif._LD, %**	3.17 ± 4.44	3.61 ± 4.71	1.45	0.985 (0.944 – 0.996)	0.561	1.048
**Abs. Dif._LD, mm**	0.20 ± 0.29	0.22 ± 0.31	0.10	0.987 (0.987 – 0.950)	0.464	0.954
**Baseline PSV, cm/s**	27.93 ± 10.44	28.02 ± 10.34	3.283	0.999 (0.994 – 0.999)	0.365	0.846
**Post-test PSV, cm/s**	57.2 ± 12.94	58.14 ± 14.61	2.581	0.999 (0.990 – 0.999)	0.95	2.633

Abs. Dif._LD, absolute differences in lumen diameter between baseline and post-test, cross – sectional area; CCA, common carotid artery; IMT, intima-media thickness; LD, lumen diameter; MDC, minimum detectable change; PSV, blood velocities at systolic peaks; Rel. Dif._LD, relative differences in lumen diameter between baseline and post-test; SEM, standard error measurement.

## 4 Discussion

### 4.1 General results

To the authors' knowledge, this is the first randomized clinical trial study to date that analyzes the immediate acute vascular effect after the application of non-invasive NESA neuromodulation compared to a Placebo group in healthy adults.

In summary, the results of this study showed that the NESA group exhibited significant increases in CSA, LD, Abs.Dif._LD, and Rel.Dif._LD of the left CCA compared to the Placebo group. Additionally, HR decreased after the session regardless of the intervention, indicating a time effect but no difference between groups.

Regarding the sociodemographic variables, no differences were determined in the descriptive variables age, height, and BMI; however, differences were observed in weight, with the Placebo group presenting lower values (66.2 ± 9.70) compared to the NESA group (73.8 ± 11.0). Although differences in weight were observed between the groups, both remained within the normal BMI range. Therefore, these differences are not deemed a confounding factor in the interpretation of our findings. However, in contexts where metabolic conditions such as diabetes or obesity are present, these variations may warrant consideration due to their association with autonomic nervous system dysregulation (Limberg et al., 2022).

### 4.2 Sonographic differences in left CCA and hemodynamics variables

The main acute effects derived from the application of a NESA vs. Placebo protocol on the vascular system were the increase in arterial CSA (*p* < 0.001), an increase in the LD of the CCA (*p* < 0.001) as well as an increase in the Abs.Dif._LD (*p* < 0.001; ES = 1.15) and Rel.Dif._LD (*p* < 0.001; ES = 1.35). The differences observed between groups before and after the NESA intervention and the Placebo may support the hypothesis that systemic stimulation through microcurrents could influence the ANS (Kolimechkov et al., 2022). Although comparable studies are limited, the review by Wang et al. (2021) highlights potential practical applications of transcutaneous stimulation of the auricular branch of the vagus nerve for cardiovascular modulation. For example, Clancy et al. (2014) showed that stimulation of the auricular branch of the vagus nerve can enhance heart rate variability and decrease skin sympathetic activity in healthy individuals. Additionally, chronic stimulation has been reported to reduce ventricular arrhythmias, left stellate ganglion activity, and sympathetic nerve remodeling in canine models following myocardial infarction (Yu et al., 2016). However, these findings must be interpreted cautiously, as our study did not involve electrode placement on the auricular branch of the vagus nerve. Furthermore, the limited sample size of our study, as well as that of other clinical studies, presents an additional challenge, as it potentially introduces biases in the interpretation and generalization of the obtained results, as well as in their extrapolation to other populations or pathological conditions.

A possible explanation for the observed increase in LD and CSA of the left CCA could be related to the increase in parasympathetic activity of the ANS. However, despite the lack of previous studies evaluating the effect of NESA on vascular responses and hemodynamic regulation, evidence from other electrical stimulation techniques, such as carotid sinus and vagus nerve stimulation (VNS), suggests their potential influence on hemodynamic variables. This rationale supports our approach in analyzing the effects of NESA based on findings from these established modalities (Gierthmuehlen and Plachta, 2015; Wang et al., 2021; Salavatian et al., 2024). In particular, electrical VNS triggers the release of acetylcholine, which activates vascular muscarinic receptors, leading to nitric oxide (NO) production. This induces smooth muscle relaxation, vasodilation, a decrease in vascular tone, an increase in vessel diameter, and improved blood flow (Schultz, 2009; Sheng and Zhu, 2018). Supporting this, Clancy et al. suggested that vagus nerve stimulation could reduce sympathetic nerve activity and enhance heart rate variability, indicating a potential shift in autonomic balance toward parasympathetic predominance (Clancy et al., 2014). Allen et al. (2022), in a study conducted on rodent models using fluorescence-based measurement techniques, observed that vagus nerve stimulation may promote NO release in the cardiac ventricle, which could be associated with cardioprotective effects.

Despite these findings, an increase in LD was also observed in the Placebo group during the assessment of the left CCA. One possible hypothesis to explain this effect could be the influence of positional changes during the assessment or intervention period, even though all participants were evaluated in the same position (Xiang et al., 2023). Additionally, participants remained in a relaxed and calm environment for approximately 40 minutes before the second assessment, which may have modulated their physiological responses (Gaertner et al., 2023). Carter et al. (2016) reported significant differences in the comparison of sexes regarding CCA shear stress in 18 healthy participants subjected to hipercapnia. Although they did not report significant sex-related differences for the variable Dif. Rel_LD (%) (mean change: 5.8 ± 3.0%), they observed a greater percentage change in women (6.2 ± 3.2%) compared to men (5.4 ± 2.9 %). Given the sex distribution in our study reported in Table 1 (Females + NESA = 57.1%; Females + Placebo = 42.9% (*n* = 21), the differences in sex distribution should be considered when interpreting these results (Yaghouby et al., 2020). However, the change observed in the Placebo group, close to 6.0%, does not appear to be attributable to the higher proportion of women in the NESA group and their greater arterial dilation response, at least based on the results from the Placebo group. This suggests that factors such as postural changes and the Placebo effect may have contributed to this outcome.

Regarding the IMT of the posterior wall of the CCA, a decrease was observed following the immediate application of NESA, compared to the Placebo. IMT adaptation is a well-established subclinical marker of cardiovascular and atherosclerotic risk, influenced by factors such as age, hypertension, and diabetes. In patients with advanced atherosclerotic plaques, an increase in the LD of the CCA has been identified as a compensatory response to IMT thickening. This mechanism maintains vascular flow despite carotid narrowing. Although the immediate effect of noninvasive electrical stimulation on IMT adaptation in healthy adults remains unexplored, Thijssen et al. (2011) demonstrated that acute changes in vascular tone, induced by the sublingual administration of glyceryl trinitrate, can result in short-term alterations in IMT emphasizing the dynamic and modifiable nature of this parameter. While the interpretation of these findings should be approached with caution, the observed changes as an adaptive compensatory mechanism of IMT to the increase in LD, likely regulated through the modulation of muscle tone, which is directly influenced by autonomic sympathetic activity (Qu and Qu, 2015).

Although immediate changes in vascular structure were observed, the direct mechanistic links between this form of neuromodulation, carotid artery morphology, and its resulting therapeutic effects remain unclear (Huffman et al., 2023; Shvartz et al., 2023; Hesampour et al., 2024). Notably, no significant differences in sBP or dBP were detected immediately after the intervention. This absence of immediate changes could reflect the complex interplay of factors regulating BP, which may buffer acute variability and transient peaks. Alternative approaches, such as transient BP assessments, might provide a more accurate and stable representation of cardiovascular dynamics (Schutte et al., 2022).

As for HR variable, both groups exhibited a reduction in values, likely influenced by the calming environment in which participants remained, with no significant group-by-time interaction detected (Gaertner et al., 2023). The results of our study differ from those reported by García et al. (2022), which indicated reductions in the lowest night-time HR, average night-time HR, and total night-time awake time following NESA stimulation compared to a Placebo in professional basketball players. Their findings were based on a 6-week intervention with twice-weekly 45-min sessions. These discrepancies could be attributed to differences in the stimulus dose, highlighting the importance of this factor when interpreting and comparing results. However, the lack of changes in hemodynamic variables despite echographic alterations remains unexplained, warranting further research into underlying mechanisms and localized hemodynamics, including carotid artery blood flow and shear stress, to evaluate the direct impacto of NESA therapy.

### 4.3 Limitations and future research

This study has several limitations. Firstly, the use of a consecutive sampling method for participant recruitment may affect the generalizability of the findings. Sex-related changes in vascular response should be considered as a limitation of the study associated with sampling and differences must be consiered for future research. Future designs incorporating analysis of vascular ultrasound variables, such as shear stress, should take this limitation into account (Carter et al., 2016; Yaghouby et al., 2020). Secondly, the evaluation of ultrasound vascular variables was not conducted using automated software for detecting changes in thickness, which may introduce measurement bias. Instead, the external program ImageJ was used to assess the thickness of the CCA. Thirdly, while the therapy involves the use of subsensory microcurrents, the effect observed in the Placebo group cannot be attributed solely to the relaxed recumbent position of the participants, nor exclusively to the relaxed environment during the application of NESA. Some study designs in transcutaneous applications propose placing the electrodes outside the territory of sensory endings to minimize expectation bias (Clancy et al., 2014; Antonino et al., 2017). Comparing different electrode placement modalities during NESA application could provide valuable insights into the true effects on the control group. Lastly, the study design focuses solely on the acute effects of the intervention, limiting the scope of the findings; additionally, the lack of a crossover design represents another limitation of the study. Future research is needed to explore the medium- and long-term therapeutic effects of non-invasive NESA neuromodulation and its relationship with hemodynamic and vascular ultrasound variables, particularly in pathological conditions such as hypertension and metabolic diseases. Incorporating ultrasound parameters, such as shear stress and blood flow, as well as the evaluation of other peripheral vascular structures, such as the brachial or popliteal arteries, may provide deeper insights into the specific effects of NESA on the autonomic nervous system and its interaction with hemodynamic variables under these pathological conditions (Salavatian et al., 2024).

## 5 Conclusions

The application of the non-invasive neuromodulation NESA protocol, compared to a Placebo, showed immediate short-term sonographic vascular modulation in healthy adults, including an increase in LD and CSA, as well as a decrease in IMT of the left CCA. However, these results should be interpreted with caution, as no differences were observed in hemodynamic variables, and the Placebo group also showed slight improvements in vascular ultrasound variables.

## Data Availability

The raw data supporting the conclusions of this article will be made available by the authors, without undue reservation.
